# Swine Pudendal Nerve as a Model for Neuromodulation Studies to Restore Lower Urinary Tract Dysfunction

**DOI:** 10.3390/ijms25020855

**Published:** 2024-01-10

**Authors:** Alice Giannotti, Stefania Musco, Vincenzo Miragliotta, Giulia Lazzarini, Andrea Pirone, Angela Briganti, Claudio Verardo, Fabio Bernini, Giulio Del Popolo, Silvestro Micera

**Affiliations:** 1 The BioRobotics Institute and Department of Excellence in Robotics and AI, Scuola Superiore Sant’Anna, 56127 Pisa, Italy; alice.giannotti@santannapisa.it (A.G.); claudio.verardo@santannapisa.it (C.V.); 2 Neuro-Urology Department, Careggi University Hospital, 50134 Firenze, Italy; muscos@aou-careggi.toscana.it (S.M.); delpopolog@aou-careggi.toscana.it (G.D.P.); 3 Department of Veterinary Sciences, University of Pisa, 56124 Pisa, Italy; vincenzo.miragliotta@unipi.it (V.M.); giulia.lazzarini@phd.unipi.it (G.L.); andrea.pirone@unipi.it (A.P.); angela.briganti@unipi.it (A.B.); 4 BioMedLab, Scuola Superiore Sant’Anna, 56127 Pisa, Italy; fabio.bernini@santannapisa.it; 5 Bertarelli Foundation Chair in Translational NeuroEngineering, Center for Neuroprosthetics and Institute of Bioengineering, École Polytechnique Fédérale de Lausanne, 1015 Lausanne, Switzerland

**Keywords:** pudendal nerve, pig animal model, lower urinary tract dysfunction, intraneural prosthesis

## Abstract

Lower urinary tract dysfunction, such as incontinence or urinary retention, is one of the leading consequences of neurological diseases. This significantly impacts the quality of life for those affected, with implications extending not only to humans but also to clinical veterinary care. Having motor and sensory fibers, the pudendal nerve is an optimal candidate for neuromodulation therapies using bidirectional intraneural prostheses, paving the way towards the restoration of a more physiological urination cycle: bladder state can be detected from recorded neural signals, then an electrical current can be injected to the nerve based on the real-time need of the bladder. To develop such prostheses and investigate this novel approach, animal studies are still required since the morphology of the target nerve is fundamental to optimizing the prosthesis design. This study aims to describe the porcine pudendal nerve as a model for neuromodulation studies aiming at restoring lower urinary tract dysfunction. Five male farm pigs were involved in the study. First, a surgical procedure to access the porcine pudendal nerve without muscle resection was developed. Then, an intraneural interface was implanted to confirm the presence of fibers innervating the external urethral sphincter by measuring its electromyographic activity. Finally, the morphophysiology of the porcine pudendal nerve at the level of surgical exposure was described by using histological and immunohistochemical characterization. This analysis confirmed the fasciculate nature of the nerve and the presence of mixed fibers with a spatial and functional organization. These achievements pave the way for further pudendal neuromodulation studies by using a clinically relevant animal model with the potential for translating the findings into clinical applications.

## 1. Introduction

The lower urinary tract (LUT) is responsible for the storage and voiding phase, relying on the coordinated activity of a storage reservoir, the urinary bladder, and an outlet, composed of the bladder neck, the urethra, and the striated muscles of the external urethral sphincter (EUS), also known as the rhabdosphincter [1,2]. The physiology of micturition, which is conserved among mammals, involves electrical impulses traveling from supraspinal pathways along with three major players: thoracolumbar sympathetic nerves (the hypogastric nerve and sympathetic chain), sacral parasympathetic nerves, and somatic nerves (the pelvic and pudendal nerve) [3]. Since the micturition cycle is controlled by complex neural mechanisms, disorders affecting either the central, the peripheral, or the autonomic nervous system can result in LUT dysfunction [4]. Symptoms of such dysfunction, such as incontinence or urinary retention, afflict over two billion individuals, profoundly impacting their quality of life due to their physical and psychosocial consequences [4,5]. The implications extend not only to humans but also to clinical veterinary care: dogs and cats experiencing LUT dysfunction may leave urine puddles in their resting spots or involuntarily dribble while moving [3]. As in humans, this poses a significant concern due to the increased susceptibility to urinary tract infections, which can greatly affect the quality of life for both the animals and their caregivers [3].

Neuromodulation strategies, which make use of transcutaneous or implanted electrodes to deliver an electrical current to the peripheral nervous system, e.g., sacral or tibial nerves, have been used in recent years as an alternative to pharmacological treatment to restore LUT control or relieve LUT symptoms [6]. Above all, pudendal nerve stimulation is a promising alternative to sacral nerve stimulation in terms of long-term efficacy and voiding control, showing a response in 93% of patients for whom sacral nerve stimulation, already approved by the Food and Drug Administration, had failed [7,8,9]. The pudendal nerve is found within the sacral region and derives from the ventral branches of the sacral nerves. Its structure is well described in humans, where it consists of three ventral rami from S2-S4 of the sacral plexus that converge adjacent to the lateral wall of the pelvic cavity. Most of its path is associated with the branches of the internal pudendal artery and vein [10]. The pudendal nerve as a whole carries sensorimotor stimuli from/to the genital and perineal area and performs a direct control of the pelvic and perineal striated musculature, such as the external anal sphincter (EAS) and EUS [10]. Thus, the role of the pudendal nerve within the micturition process is mainly to support contraction of the EUS through cholinergic receptors responding to the somatic efferent function of the nerve when storage of urine is needed [11]. However, the existence of an afferent pathway within the pudendal nerve has also been proven: activation of the so-called ‘pudendal-to-bladder’ reflex can either evoke bladder contraction or relaxation depending on the frequency of stimulation [12].

The presence of both sensory and motor fibers makes the pudendal nerve an optimal candidate for the use of a bidirectional intraneural prosthesis: after placing the prosthesis within nerve fascicles, bladder states can be detected from sensory signals while the stimulation of motor fibers can be delivered based on the real-time need of the bladder, paving the way towards the restoration of a more physiological urination cycle [13]. However, pudendal nerve stimulation is currently performed by using epineural electrodes placed beside the nerve, which lack selectivity in nerve fiber recruitment and overall nerve stimulation may cause side effects [8,9].

To develop custom bidirectional intraneural prostheses and to investigate this novel approach, animal studies are still required since the morphology of the target nerve is fundamental to optimizing the prosthesis design. However, Shen et al. underlined the importance as well as the lack of an ideal animal model for the study of urinary bladder function [14]. Concerning the pudendal nerve, its morphology, path, and sensorimotor components were well described in male and female rats [15,16,17], female rabbits [18], and male and female cats [19,20]. Common features include its origin from the lumbosacral region and a branching pattern as it enters the sacral plexus: while in humans, a tripartite branching in a genital, a perineal, and a rectal component is described [21]. In the previously mentioned animal models, two main branches, a motor and a sensory component, appear to leave the plexus and enter the ischio-rectal fossa. Selective stimulation of the pudendal nerve’s sensory branch proved to induce bladder contraction and increase voiding efficacy. However, selectivity was achieved from surgical exposure of the sensory branch, which translates poorly in humans. [7,22]. The use of intraneural prostheses as a tool for bidirectional communication with pudendal motor and sensory pathways has also been explored using Utah arrays as neural prostheses proving their selectivity in recording genitourinary-related function in cats and controlling the EUS in dogs, but their high invasiveness makes them very traumatic, especially when implanted within the peripheral nervous system [23,24]. Larger animals such as pigs offer more comprehensive data compared to smaller rodents, thereby providing valuable insights into clinical physiology having anatomical, size, and urinary system characteristics very similar to those of humans, compared to smaller animals such as rats, rabbits, and cats [25,26]. Pigs are particularly useful for research on the initiation of micturition, as micturition patterns observed in female pigs mimic those of humans, including urethral relaxation, which is significantly different from that observed in rats and cats [25,27]. The porcine pudendal nerve is an ideal candidate, but knowledge about its morphology, functional organization, and branching pathway is limited. Studies involving porcine pudendal nerve to deal with LUT dysfunction have been limited to the use of laparoscopic surgeries for the implantation of commercial epineural tined lead electrodes [28,29]. This approach may result in abdominal viscera damage and difficulties in proper electrode fixation, as reported by Keller et al. [29]. The use of intraneural electrodes in pigs’ pudendal nerve has been limited in showing the possibility of detecting bladder fullness from pudendal neural signals [13].

Here, we present anatomical observations on the porcine pudendal nerve from dissection to histological and immunohistochemical evaluation. The aim is to identify a minimally invasive surgical approach to target the pudendal nerve fascicles responsible for EUS control and to identify the spatial distribution of the nerve’s motor and sensory fibers. The size of the pudendal nerve, the number of fascicles, fascicle distribution, and fiber function are indeed fundamental for the development of highly selective customized intraneural prosthesis. These results pave the way for neuromodulation studies on large animal models to treat urinary bladder dysfunction and restore the physiological urination cycle.

We can summarize the contribution of this study with the following list:

Describing the surgical procedure to implant an intraneural interface within the porcine pudendal nerve without muscle resection and confirmation of the EUS recruitment with neuromodulation studies;

Describing the branching, and the morphophysiology of the porcine pudendal nerve at the level of surgical exposure.

## 2. Results

The surgical approach to target the pudendal nerve fibers with a minimally invasive procedure in pigs was identified and described in Section 2.1. The exposed region was confirmed to give rise to the pudendal nerve by applying electrical stimulation with the use of an intraneural electrode, described in Section 2.2, during EAS and EUS electromyographic (EMG) activity monitoring. Morphological and functional characterization were performed on the previously mentioned target area and described in Section 2.3 and Section 2.4, confirming the fasciculated and mixed nature of the pudendal nerve.

### 2.1. Surgical Site and Pudendal Nerve Gross Anatomy

Ischio-rectal surgery was performed as described in Section 4.1. The surgically exposed portion was identified as the rostral one of the two spinal origins. It was possible to validate its identification since its mechanical or electrical stimulation led to the visible contraction of the EAS. The rostral origin was selected as the surgical best option for neuromodulation studies and neural prosthesis implantation since it did not require muscle and ligament resection.

Post-sacrifice dissection allowed the sampling of all pudendal nerve origins and branches: the swine pudendal nerve consisted of two sacral origins, a common segment, and two main branching segments (a cutaneous and a splanchnic one).

The rostral sacral origin was identified as departing from S1 and lying between the sciatic nerve and the pudendal vein and artery.

The caudal sacral origin was identified as departing from S2 and is mostly covered by the thoracolumbar fascia.

The common segment travels caudally and is covered by the sacrospinous ligament.

The cutaneous branch gives rise to 2–3 individual nerves.

The splanchnic branch also originates 2–3 individual nerves.

**Figure 1:**
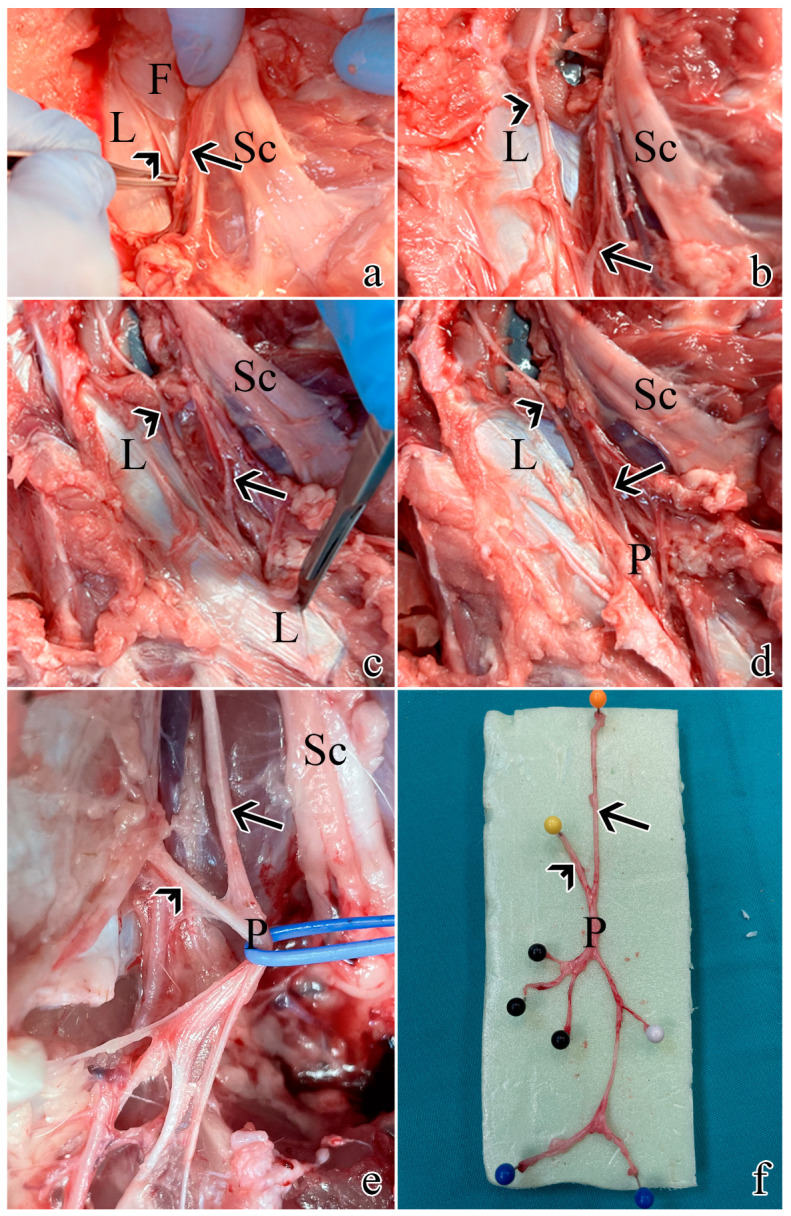
Post-sacrifice dissection of all pudendal nerve origins and branches: (**a**) exposure of S1 as described in Section 4.1. using muscle- and ligament-sparing transgluteal surgery; (**b**) the thoracolumbar fascia was removed rostrally and caudally to expose S2; (**c**) caudal dissection allowed the identification of the sacrospinous ligament; (**d**) resection of the ligament allowed visualization of the proper pudendal nerve; (**e**) the pudendal nerve gives rise of multiple branches; (**f**) a pudendal nerve sample with its S1 and S2 origins and branches: the orange pin highlights S1 origin, the yellow pin highlights S2 origin, the black pins highlight the splanchnic component, while the blue and white pins the cutaneous ones. Arrows highlight S1, while arrowheads indicate S2. L = sacrospinous ligament; F = thoracolumbar fascia; Sc = sciatic nerve; P = pudendal nerve.

These findings are summarized in Figure 1.

### 2.2. Intraneural Electrode Implantation and Electrical Stimulation

Preliminary neuromodulation studies were performed to prove the possibility of implanting a functional intraneural electrode using the surgical approach described in detail in Section 4.1. This made it possible to confirm the recruitment of neural fibers controlling the perineal area, particularly the EAS and the EUS, in the identified location.

**Figure 2:**
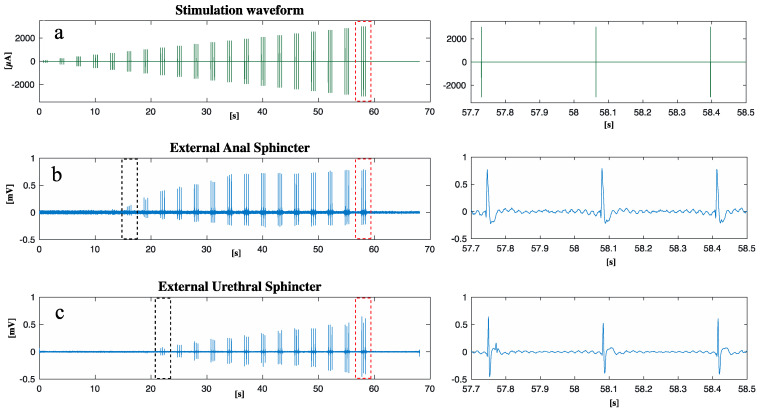
After the implantation of an intraneural prosthesis, an electrical current was delivered to S1 while the EAS and EUS EMG activity was measured: (**a**) waveform of the stimulation current delivered; (**b**) EAS EMG response to stimulation; (**c**) EUS EMG response to stimulation. A close-up of the region marked with red dots can be found on the right, respectively, for (**a**), (**b**), and (**c**). The black dots in (**b**,**c**) show the first contraction of the EAS and EUS, respectively.

Figure 2 shows the stimulation paradigm applied (Figure 2a) to the nerve and the EMG responses of the EAS (Figure 2b) and EUS (Figure 2c). The minimum current to measure a sustained EAS contraction was 860 µA (the black dots in Figure 2b), while for EUS it was 1170 µA (the black dots in Figure 2c). The EAS reached the maximum contraction when the current amplitude was 1200 µA: a further increase of the current did not increase EAS contraction. This result was not observed for the EUS, thus the needed current to reach the maximum EUS contraction was higher than the 2 mA current applied during this study.

### 2.3. Histology

**Figure 3:**
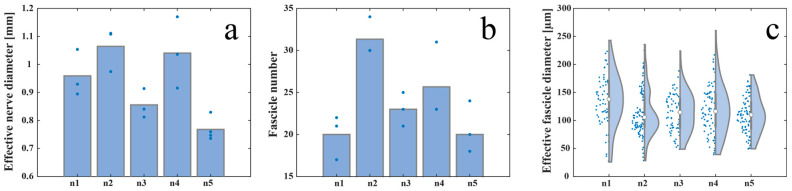
Inter-animal variability measured as (**a**) effective nerve diameter, (**b**) fascicle number, and (**c**) effective fascicle diameter among five animals. The white circles in (**c**) represent the median value and the grey bars represent the interquartile range of the previously mentioned parameters. Blue dots in (**a**,**b**) represent the absolute value of the previously mentioned parameters.

The effective diameter of the S1 left pudendal nerve ranged from a minimum value of 768 ± 42 µm and a maximum value of 1065 ± 78 µm for the five male far pigs considered in this study, as shown in Figure 3a. S1 appeared to be composed of a number of fascicles ranging from 20 ± 3 to 31 ± 2 (Figure 3b), showing distinct perineuria and having an effective diameter varying between 109 ± 33 µm and 135 ± 49 µm (Figure 3c).

**Figure 4:**
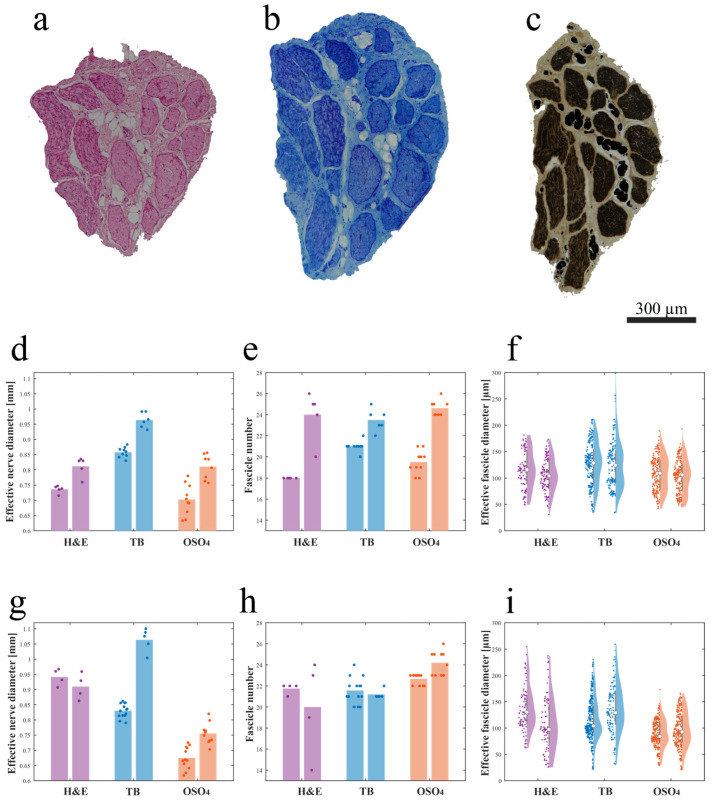
Intra-animal variability measured by serially sectioning and processing with three different methods (**a**–**c**), the left S1 pudendal nerve effective nerve diameter, fascicle number, and effective fascicle diameter (**d**–**f**) and right S1 pudendal nerve effective nerve diameter, fascicle number and effective fascicle diameter (**g**–**i**): (**a**) example of the H and E-stained paraffin-embedded section; (**b**) example of the TB-stained JB4-embedded section; (**c**) example of the OSO_4_-post-fixed JB4-embedded section; (**d**) effective nerve diameter, (**e**) fascicle number, and (**f**) effective fascicle diameter for the left pudendal nerve; (**g**) effective nerve diameter, (**h**) fascicle number, and (**i**) effective fascicle diameter for the right pudendal nerve (the white circles in (**f**,**i**) represent the median value and the grey bars in (**d**,**e**,**g**,**h**) represent the interquartile range of the previously mentioned parameters). Purple, blue, and orange dots in (**d**-**i**) represent the absolute value of the previously mentioned parameters obtained for H and E-stained paraffin-embedded sections, TB-stained JB4-embedded sections, and the OSO_4_-post-fixed JB4-embedded sections respectively.

A prevalence of big, myelinated fibers was observed when the S1 left pudendal nerve was processed with Hematoxylin and Eosin (H and E) procedure (Figure 4a), Toluidine Blue (TB) procedure (Figure 4b), and osmium tetroxide (OSO_4_) post-fixation (Figure 4c). When S1 was serially sectioned, the mean effective diameter did change craniocaudally. Comparing blocks with the same staining (1–4, 2–5, 3–6), the effective nerve diameter showed an increasing trend with *p* < 0.01 (Figure 4d,g), except for blocks 1–4 of the right nerve where no significance was found. In the latter case, the number of fascicles seems to decrease, but the standard deviation was high (Figure 4g). Since the number of fascicles in block 3 also appears to be greater than in block 4 (Figure 4h), despite its caudal location, part of the sample may have been damaged during the sectioning procedure and some of the branches may have been lost. For the left nerve, this growth was given by both a progressive increase in the number of fascicles, with *p* < 0.01 (Figure 4e), and an increase in their effective diameter, with *p* < 0.1 (Figure 4f). For the right nerve, the growth of the effective nerve diameter can be attributed to the significant increase in the diameter of fascicles that showed *p* < 0.001, except for blocks 1–4 where, however, the violin plot is expanded, thus the standard deviation is high (Figure 4i). The number of fascicles in the osmium tetroxide blocks showed a significant increase, while the significance was not found for 1–4, 2–5 where, however, the standard deviation is high (Figure 4h).

The nerve effective diameter appeared to change when the three processing procedures were applied; in particular, blocks post-fixed in osmium tetroxide resulted in a smaller area compared to blocks embedded in paraffin and JB4 (Figure 4d,g). More specifically, comparing blocks 1–3–5, the effective diameter of the nerve in block 5 was found to be the smallest (significantly smaller than 3, with *p* < 0.001) despite being positioned more caudally. Similarly, for blocks 2–4–6, the effective diameter of the nerve in block 6 was found to be the smallest (significantly smaller than 4 with *p* < 0.001). Post-fixation in osmium tetroxide seems to cause tissue shrinking. This hypothesis would be supported by the fact that there is not such an evident decrease in the number of fascicles and/or their diameter.

### 2.4. Immunohistochemistry

Choline Acetyltransferase (ChAT), Tyrosine hydroxylase (TH), and Substance P (SP) were chosen as neuronal markers to investigate the sensorimotor composition of the S1 tract. ChAT, by catalyzing the transfer of an acetyl group from the coenzyme acetyl-CoA to choline, yielding acetylcholine, will unequivocally identify somatic motoneurons-derived fibers in S1 and/or parasympathetic preganglionic fibers; TH was instead selected as a marker of postganglionic sympathetic fibers since it catalyzes the conversion of L-tyrosine into L-3,4-dihydroxyphenylalanine which is a precursor of dopamine, norepinephrine, and epinephrine; Substance P is instead a marker of sensory neurons.

**Figure 5:**
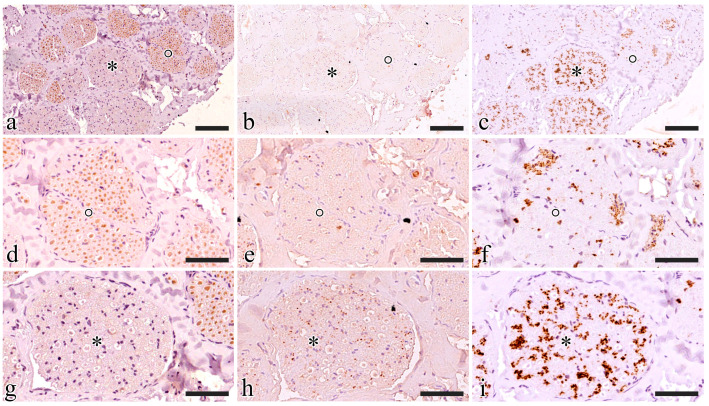
Functional characterization of the S1 pudendal nerve tract by evaluating the immunoreactivity to ChAT (**a**,**d**,**g**), SP (**b**,**e**,**h**), and TH (**c**,**f**,**i**). (**a**–**c**) Show the same region exposed to ChAT, SP, and TH, respectively; two fascicles were highlighted with a circle and an asterisk, then magnification of the highlighted fascicles was shown in (**d**–**f**) and (**g**–**i**) respectively; (**d**–**f**) show the fascicle highlighted with a circle exposed to ChAT, SP, and TH, respectively; (**g**–**i**) show the fascicle highlighted with an asterisk exposed to ChAT, SP, and TH, respectively. The scale bars are equal to 100 µm in (**a**–**c**) and 50 µm in (**d**–**i**).

S1 generally showed a prevailing ChAT-immunoreactive area while the remaining portion was TH positive (Figure 5a–c). ChAT immunoreactivity was estimated at around 75% of the nerve in all five animals.

ChAT-immunoreactive fascicles also displayed some TH-positive axons and never showed SP-positive elements. TH-positive axons included in ChAT-immunoreactive fascicles were always segregated in specific areas where ChAT fibers were completely lacking (Figure 5d–f).

TH immunoreactivity prevailed in ChAT-immunonegative fascicles where SP-positive small axons were also visible. SP was only present in TH-immunoreactive areas (Figure 5g–i).

## 3. Discussion

Previous studies of the literature highlighted the need for animal models for developing innovative strategies to treat LUT dysfunction [14]. Thor et al. emphasized the importance of investigations into the afferent and efferent neurons innervating perineal muscles in large animal species [2]. Pigs offer the advantages of having anatomical, size, and urinary system characteristics very similar to those of humans, thus providing results more easily translated into clinical research [25].

The pudendal nerve anatomy in the swine species was found to be poorly described, beside some general descriptions found in veterinary textbooks [30], it was not possible to find a detailed description of a standardized approach to surgically target the pudendal nerve, nor an extensive characterization of its branching pattern, morphology, and functional anatomy. It was thus necessary to work on dead animals to precisely describe the nerve path and thus generate surgical access to the pudendal nerve. An ischio-rectal approach was adopted since it reduces the risk of abdominal viscera damage [18]. First, we approached the sciatic nerve as described by Strauss et al. [31]; then rostrally following its course towards the spine, it was possible to see its shared emergence from the sacral spinal nerves with the pudendal nerve. The pudendal nerve was identified as lying near the pudendal vein and artery. Once the nerve was identified, muscle and ligament-sparing surgical access was fined-tuned as described in Section 4.1. The S1 pudendal spinal origin was the only minimally invasively approachable segment. Indeed, in mammals, the pudendal nerve generally arises from the spinal nerves in the lumbosacral region, forming a sacral plexus in the pelvic region. It then enters the pelvic cavity and originates as the proper pudendal nerve, which lies beside the sacrospinous ligament. At the level of the ischio-rectal fossa, it then branches off to go to the perineal muscles (the EUS, EAS, bulbospongious muscle, and ischiocavernous muscle) [11]. The ischio-rectal approach for pudendal neuromodulation has been described in other animal models, mainly rats, and cats. However, the described incision was created in close proximity to the base of the tail, and both muscle and fat were excised [7]. The resection of gluteal muscles makes the clinical translation of this study much more difficult. Therefore, surgically accessing the proper pudendal nerve and creating a space for the implantation of a neuroprosthesis in a muscle and ligament-sparing approach is not possible. We thus decided to target the S1 pudendal spinal origin as the site for the implantation of neural prostheses. It is worth noting that, as highlighted by Barone et al. [30], the anatomical variability between species and animals is very wide, so the S1 target identified in this study as the most cranial sacral root could emerge from other sacral vertebrae in some cases. Stimulation of S1, described in Section 4.2, showed that at this location it is possible to target the EUS motor fiber. Giannotti et al. [13] had already indirectly shown that this segment also contains sensory fibers from which it is possible to estimate the bladder state. Thus, this surgical approach could be an ideal candidate for the implantation of a bidirectional intraneural prosthesis enabling selective targeting of motor and sensory fibers. Selectivity in engaging neural fibers was demonstrated to be highly desirable to restore continence, increase voiding efficacy, and reduce possible side effects such as detrusor-sphincter-dyssynergia, but so far this was achieved by implanting multiple neural prostheses in pudendal nerve sensory and motor branches [7,22,32]. The implantation of a single bidirectional prosthesis could reduce tissue damage and surgical complications.

Once the implantation site was identified, we attempted to histologically characterize the S1 pudendal spinal origin in its morphology. This will allow the future development of custom neural prostheses based on the nerve dimensions and morphology. Foditsch et al., 2014, previously attempted to histologically characterize the pudendal nerve, reporting a highly variable nerve diameter ranging from 0.2 to 1.1 mm [28]. However, these results are poorly comparable to those shown here since their access to the pudendal nerve had been performed in laparoscopy, thus approaching the nerve within the perineal region. Furthermore, their animal size was lower and females instead of males were used; thus, it is reasonable that our findings show a higher nerve diameter, with a minimum value of around 0.8 mm. Generally, previous studies have focused efforts on characterizing specific pudendal nerve branches: Bremer et al. histologically characterized the motor and the sensory branch of the pudendal nerve in rats [33]; the motor branch in female rats has been also analyzed by Kane et al. [34], but the characterization of the proper pudendal nerve before its branching has not been reported in rats. An extensive characterization of the dorsal nerve of the penis, a branch of the pudendal nerve, was reported in cats, but these results are not comparable to those described here for the different locations examined [19]. A histological section of pudendal nerve fascicles has also been reported in monkeys but the sample was explanted from the pelvic region instead of the ischio-rectal region [35]. Finally, a deep analysis of the human pudendal nerve was described by Gustafson et al. [21], but in this case the focus has also been directed at the nerve branches instead of the sacral spinal nerves that originate the pudendal nerve. This work represents the first extensive description of the porcine pudendal nerve at the level of the S1 pudendal spinal origin, which can be targeted with muscle and ligament-sparing surgery. We reported an intra-animal and inter-animal characterization of the S1 tract, estimating the parameters of effective nerve diameter, effective fascicle diameter, and fascicle number. The diameter of the nerve was found to increase progressively in the caudal direction, due to both progressive branching of the nerve and increasing fascicle diameter. In each case, a high standard deviation demonstrates the high anatomical variability making it difficult to extrapolate conclusions. The staining method chosen also seems to have an impact on calculating the actual value of nerve size. However, the parameters obtained allow for indications regarding the average number of fascicles and the size range of the S1 tract of the porcine pudendal nerve, paving the way for optimized sizing of ad hoc developed neural prostheses.

Micturition, as reported above, is, however, a complex physiological process where the pudendal nerve not only controls the sphincteric contraction and relaxation of the EUS but has been shown to indirectly take part in bladder contraction or relaxation throughout the ‘pudendal-to-bladder’ reflex [12]. Thus, the functional phenotype of pudendal nerve fibers and their spatial distribution were investigated using selected antibodies (TH, ChAT, P-substance). In the current study, S1 pudendal spinal origin was found to be a mixed nerve where somatic and autonomic neurotransmitters are present, confirming also Timoth et al.’s fundings in pigs, in contrast to the prevailing belief that it is purely somatic [36]. While we can assume that TH-immunoreactive fibers do belong to the sympathetic system, we cannot conclude that ChAT-positive fibers also include parasympathetic preganglionic fibers that might belong to the pelvic nerve which also originate from S1. However, the probability that they are preganglionic pelvic fibers is not high since, in both rats and rabbits, it has been seen that the pelvic nerve immediately branches from S1, entering the pelvic plexus within the pelvic cavity, while the pudendal nerve and sacral plexus lie on the dorsal surface of the ischium to enter the pelvic cavity within the ischio-rectal fossa [15,18]. The presence of TH-positive fibers in pigs highlights the role of the pudendal nerve: besides the sensory and sensorimotor innervation of the striated perineal muscles, this has also been shown to provide sympathetic postganglionic innervation to the pelvic organs [15]. More precisely, sympathetic innervation has been hypothesized to be responsible for the innervation of the penis and the control of the genitourinary system in rats [37,38,39].

Electrical stimulation of the S1 pudendal spinal origin resulted in EUS contraction, confirming that this location is suitable for pudendal neuromodulation experiments aiming at restoring lower urinary tract dysfunction. The EAS has been shown to have a lower activation threshold compared to the EUS and reaches its maximum contraction level at lower current amplitudes. The fact that the EAS is activated for lower currents suggests the need to use stimulation that selectively activates only EUS. This is necessary in order to modulate EUS activity to restore urinary dysfunction without inducing problems such as fecal incontinence. For this reason, the use of an intraneural prosthesis could provide the required selectivity, as already shown in human upper limb amputees [40]. A limitation of this preliminary study is that the electrical contacts of the intraneural electrode implanted were all activated simultaneously, making it impossible to test the ability to be selective toward EUS. Future studies will focus on stimulating one site at a time in order to find contacts that selectively target the fibers responsible for controlling the EUS. In addition, future studies will aim to understand how to translate the results of this study, performed on S1 in pigs, into humans, where the pudendal nerve originates at S2-S4, with a major contribution from S3.

Our results showed a spatial separation between the TH- and SP-positive fibers and the ChAT-positive fibers, suggesting a spatial organization of fascicles mainly responsible for somatic control of perineal muscles (ChAT positive areas) and fascicles mainly responsible for carrying sensory information from the pelvic area and autonomic control of genital functions. This funding will open the possibility for further neuromodulation studies targeting the pudendal nerve at the S1 level with minimally invasive implanted bidirectional intraneural prostheses, which could selectively interact with the functionally different areas of the pig pudendal nerve, thus enabling both the monitoring of bladder state from sensory fibers and the control of the sphincters with motor ones.

## 4. Materials and Methods

### 4.1. Surgical Site and Pudendal Gross Anatomy

The surgical procedure was defined by using three male animals unrelated to this study that were dissected to identify the pudendal sacral spinal nerves and their accessibility. The rostral S1 pudendal spinal origin was selected as the implantation site for the neuromodulatory experiments.

**Figure 6:**
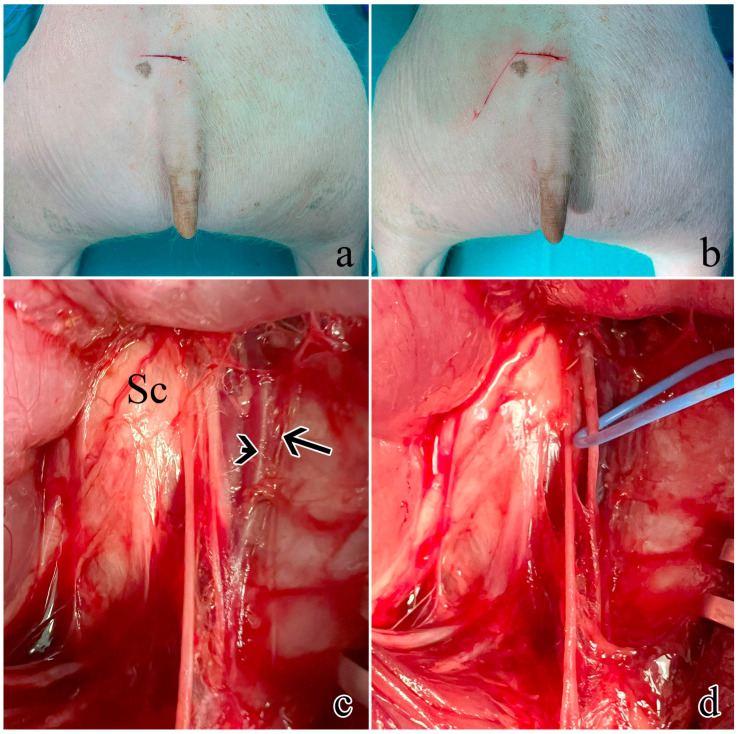
Surgical procedure to expose the rostral S1 pudendal spinal origin: (**a**) cutaneous incision on a transversal plane; (**b**) second obliquus cutaneous incision; (**c**) exposure and retraction of the gluteus superficialis and medius. The arrow indicates the pudendal artery, the arrowhead shows the pudendal vein, while Sc is the sciatic nerve; (**d**) the pudendal nerve was identified medially lining the internal pudendal artery and vein and highlighted with a blue marker.

Five male farm pigs (Azienda Agricola e Allevamento Alessandro Stassano, Cedri di Peccioli Pisa) weighing 30–35 kg were used. The animals received premedication with Zoletil^®^ at a dosage of 10 mg/kg. Subsequently, they were induced into anesthesia through the intravenous administration of Propofol at a dosage of 2 mg/kg, followed by maintenance under 1–3% sevoflurane in a mixture of air containing 50% oxygen. Throughout the procedure, continuous monitoring was employed to track oxygen saturation, arterial pressure, and heart rate. Surgical access was performed to expose the left pudendal nerve and a first cutaneous incision was made on a transversal plane located 6 cm rostrally from the base of the tail, 2 cm wide from the sagittal plane (Figure 6a); a second cutaneous incision was performed starting from the lateral end of the first one going caudally and laterally at a 30° angle (Figure 6b), ideally following the separation between the muscles gluteus superficialis and medius; after the exposure of the gluteal fascia, the gluteus superficialis and medius were retracted to expose the sciatic nerve (Figure 6c). Once the sciatic nerve was found, the pudendal one was identified medially lining the internal pudendal artery and vein (Figure 6d).

### 4.2. Intraneural Electrode Implantation and Electrical Stimulation

Once the rostral pudendal sacral spinal nerve was identified, an intraneural electrode was implanted within nerve fascicles. The design of the custom-developed intraneural electrode was inspired by the Transversal Intrafascicular Multichannel Electrode (TIME) [41]. The intraneural electrode was composed of 16 electrical contacts coated with iridium oxide (IrOx) lying on a flexible polyimide substrate. A detailed description of the design and fabrication procedure can be found in a previous study by our group [13], while the insertion procedure was described by Boretius et al. [41]. Briefly, the intraneural electrode was linked to a suture needle, which was threaded within the nerve fascicles; the needle was then pulled from the opposite side of the nerve, allowing the electrical contacts to interface with the nerve fascicles. The electrode was then connected to a TDT system (Tucker-David Technologies Inc., Alachua, FL, USA) to provide the nerve with an electrical current injected through the electrical contacts. The electrical current waveform was composed of biphasic cathodic first stimuli, with increasing current amplitude ranging from 100 to 3000 µA, delivered in 20 steps with three repetitions for each current amplitude and a 200 µs pulse width (Figure 2a). Activation of the external anal sphincter was visually monitored, to confirm correct identification of the pudendal nerve.

In one animal, the activation of the external anal sphincter and the external urethral sphincter was also quantified by measuring their EMG activity. The EMG of the external anal sphincter was measured by placing needle electrodes (SpesMedica, Genova, Italy) at 9 and 3 o’clock as described by Keung [42]. The external urethral sphincter was implanted with needle electrodes (SpesMedica, Genova, Italy), after performing a midline ventral incision above the pubic bone to expose the muscle.

### 4.3. Histology

After humane sacrifice, the pudendal nerve was dissected by recovering its roots and branches, placed on a polystyrene sheet, and fixed in 10% buffered formalin.

The S1 pudendal spinal origin nerve was deeply characterized for its morphology and fibers’ immunophenotype. Formalin-fixed samples were routinely processed for either paraffin or JB-4 resin embedding. From the 5 animals (n1, n2, n3, n4, n5), both nerves were isolated and sampled.

From one animal (n5), the S1 segment from both the left and right pudendal nerve was embedded in 6 consecutive 3 mm-long blocks and then underwent three different processing procedures (in pairs) as follows. Blocks 1 and 4 were paraffin-embedded and stained following a standard H and E procedure; blocks 2 and 5 were JB4-embedded and stained with TB; blocks 3 and 6 underwent OSO_4_ post-fixation before JB4 embedding. All the blocks were serially sectioned to analyze the S1 pudendal nerve segment changes within the same animal. Blocks were cut using a microtome into 5 μm thick sections 100 μm apart. A total of 87 images of the histological sections of the S1 pudendal nerve were obtained, including 18 H and E sections, 32 TB sections, and 37 OSO_4_ sections. The parameters evaluated were the number of fascicles, the effective fascicle diameter, and the effective nerve diameter, where effective means the diameter of the equivalent circle having the same area of the fascicles or nerve, respectively. This procedure also made it possible to measure changes related to the sample processing procedure. Measures were obtained by using a custom algorithm implemented in MATLAB (version 2023b, MathWorks Inc., Natick, MA, USA) after semi-automatic segmentation of the histological images using ImageJ software (version 1.54e, National Institutes of Health, Bethesda, MD. USA), where nerve and fascicle contours were highlighted. Statistical analysis was performed by applying a Kolmogorov–Smirnov test using MATLAB (version 2023b, MathWorks Inc., Natick, MA, USA).

Samples obtained from the left pudendal nerve of the 4 remaining animals were cut into three blocks and paraffin-embedded. Three sections per block were stained with Mallory trichrome to measure the number of fascicles, effective fascicle diameter, and effective nerve diameter.

### 4.4. Immunohistochemistry

After paraffin embedding and sectioning, tissues were rehydrated for the immunostaining: epitope retrieval was carried out at 120 °C in a pressure cooker for 3 min with a Tris/EDTA buffer (pH 9.0); peroxidase quenching was carried out by incubation with 1% H_2_O_2_ in Phosphate Buffered Saline (PBS) for 7 min at room temperature (RT). Non-specific binding was prevented by incubating slides in a blocking solution composed of 0.05% Triton X (TX)-100 and 2% bovine serum albumin (SP-5050; Vector Laboratories, Newark, NJ, USA) for 1 h at RT. The slides were then incubated with either goat polyclonal anti-ChAT antibody (AB144P; Merck & Co., Inc., Readington, NJ, USA; 1:100), rabbit polyclonal anti-TH antibody (SC14007, Santa Cruz Biotechnology, 1:200), or rat monoclonal anti-SP antibody (GTX38990; Gentex Corporation, New York, NY, USA; 1:200) overnight at 4 °C. Then, the slides were rinsed in PBS (3 × 10 min), and incubated with a biotinylated anti-goat (for ChAT, BA-9500, Vector Labs) or anti-rabbit (for TH, BA-1100, Vector Labs) or anti-rat IgG antibody (for SP, BA-9400, Vector Labs). The sections were again rinsed in PBS (3 × 10 min) and incubated for 30 min with an ABC complex (PK-2100; Vector Laboratories, Newark, NJ, USA) at RT. Staining was visualized by incubating sections in diaminobenzidine solution (SK-4105; Vector Laboratories, Newark, NJ, USA). Before dehydration and mounting, the slides were counterstained with Hematoxylin for 7 s to stain the nuclei. The specificity of immunohistochemical staining was tested by replacing the primary antibodies with PBS. Under these conditions, staining was abolished. Whole slide images were acquired with Nano Zoomer Hamamatsu slide scanner at a magnification of 20× with automatic focusing.

The fascicles were scored for ChAT immunoreactivity, assuming that negative fascicles were mostly TH immunoreactive. The scores ranged from a minimum of 1, corresponding to 25% of ChAT positivity, and 4 corresponding to 100% of ChAT positivity.

## 5. Conclusions

In this study, the pig pudendal nerve was investigated with the aim of developing an animal model for neuromodulation studies to restore lower urinary tract dysfunction with a high possibility of translating the results into use in a clinical setting. We described the surgical access to safely target the main sacral spinal nerve from which the pudendal nerve originates, confirming through electrical stimulation the presence of fibers innervating the external urethral sphincter. We also characterized this nerve from the morphological and immunohistochemical point of view, confirming the highly fasciculate nature of the nerve and its spatial and functional organization. The studied location is therefore a potential candidate for further pudendal neuromodulation studies in which the use of an intraneural prostheses could enable to restore the physiological urination cycle.

## Data Availability

The data presented in this study are available on request from the corresponding author.

## References

[1] De Groat W.C., Griffiths D., Yoshimura N., Terjung R. (2014). Neural Control of the Lower Urinary Tract. Comprehensive Physiology.

[2] Thor K.B., De Groat W.C. (2010). Neural Control of the Female Urethral and Anal Rhabdosphincters and Pelvic Floor Muscles. Am. J. Physiol. Regul. Integr. Comp. Physiol..

[3] Gernone F., Uva A., Maiolini A., Zatelli A. (2022). A Review of the Neural Control of Micturition in Dogs and Cats: Neuroanatomy, Neurophysiology and Neuroplasticity. Vet. Res. Commun..

[4] Panicker J.N. (2020). Neurogenic Bladder: Epidemiology, Diagnosis, and Management. Semin. Neurol..

[5] Zhang A.Y., Xu X. (2018). Prevalence, Burden, and Treatment of Lower Urinary Tract Symptoms in Men Aged 50 and Older: A Systematic Review of the Literature. SAGE Open Nurs..

[6] Burks F.N., Bui D.T., Peters K.M. (2010). Neuromodulation and the Neurogenic Bladder. Urol. Clin. N. Am..

[7] Hokanson J.A., Langdale C.L., Sridhar A., Milliken P., Grill W.M. (2021). State-Dependent Bioelectronic Interface to Control Bladder Function. Sci. Rep..

[8] Spinelli M., Malaguti S., Giardiello G., Lazzeri M., Tarantola J., Hombergh U.V.D. (2005). A New Minimally Invasive Procedure for Pudendal Nerve Stimulation to Treat Neurogenic Bladder: Description of the Method and Preliminary Data. Neurourol. Urodyn..

[9] Peters K.M., Feber K.M., Bennett R.C. (2005). Sacral versus Pudendal Nerve Stimulation for Voiding Dysfunction: A Prospective, Single-Blinded, Randomized, Crossover Trial. Neurourol. Urodyn..

[10] Kinter K.J., Newton B.W. (2023). Anatomy, Abdomen and Pelvis, Pudendal Nerve. StatPearls.

[11] Furness J.B. (1999). The Autonomic Nervous System and Its Effectors by Alison Brading. Trends Neurosc..

[12] Woock J.P., Yoo P.B., Grill W.M. (2011). Mechanisms of Reflex Bladder Activation by Pudendal Afferents. Am. J. Physiol. Regul. Integr. Comp. Physiol..

[13] Giannotti A., Lo Vecchio S., Musco S., Pollina L., Vallone F., Strauss I., Paggi V., Bernini F., Gabisonia K., Carlucci L. (2023). Decoding Bladder State from Pudendal Intraneural Signals in Pigs. APL Bioeng..

[14] Shen J.-D., Chen S.-J., Chen H.-Y., Chiu K.-Y., Chen Y.-H., Chen W.-C. (2021). Review of Animal Models to Study Urinary Bladder Function. Biology.

[15] McKenna K.E., Nadelhaft I. (1986). The Organization of the Pudendal Nerve in the Male and Female Rat. J. Comp. Neurol..

[16] Pacheco P., Camacho M.A., García L.I., Hernández M.E., Carrillo P., Manzo J. (1997). Electrophysiological Evidence for the Nomenclature of the Pudendal Nerve and Sacral Plexus in the Male Rat. Brain Res..

[17] Pacheco P., Martinez-Gomez M., Whipple B., Beyer C., Komisaruk B.R. (1989). Somato-Motor Components of the Pelvic and Pudendal Nerves of the Female Rat. Brain Res..

[18] Cruz Y., Hernández-Plata I., Lucio R.A., Zempoalteca R., Castelán F., Martínez-Gómez M. (2017). Anatomical Organization and Somatic Axonal Components of the Lumbosacral Nerves in Female Rabbits. Neurourol. Urodyn..

[19] Mariano T.Y., Boger A.S., Gustafson K.J. (2008). The Feline Dorsal Nerve of the Penis Arises from the Deep Perineal Nerve and Not the Sensory Afferent Branch. Anatom Histol. Embryol..

[20] Kawatani M., Nagel J., De Groat W.C. (1986). Identification of Neuropeptides in Pelvic and Pudendal Nerve Afferent Pathways to the Sacral Spinal Cord of the Cat. J. Comp. Neurol..

[21] Gustafson K.J., Zelkovic P.F., Feng A.H., Draper C.E., Bodner D.R., Grill W.M. (2005). Fascicular Anatomy and Surgical Access of the Human Pudendal Nerve. World J. Urol..

[22] Yoo P.B., Woock J.P., Grill W.M. (2008). Bladder Activation by Selective Stimulation of Pudendal Nerve Afferents in the Cat. Exp. Neurol..

[23] Mathews K.S., Wark H.A.C., Warren D.J., Christensen M.B., Nolta N.F., Cartwright P.C., Normann R.A. (2014). Acute Monitoring of Genitourinary Function Using Intrafascicular Electrodes: Selective Pudendal Nerve Activity Corresponding to Bladder Filling, Bladder Fullness, and Genital Stimulation. Urology.

[24] Wark H.A.C., Dowden B.R., Cartwright P.C., Normann R.A. (2011). Selective Activation of the Muscles of Micturition Using Intrafascicular Stimulation of the Pudendal Nerve. IEEE J. Emerg. Sel. Top. Circuits Syst..

[25] Dalmose A.L., Rijkhoff N.J.M., Andersen I.S., Stefania D., J‘rgensen T.M., Djurhuus J.C. (2002). Bladder and Urethral Responses to Pelvic Nerve Stimulation in the Pig. Scand. J. Urol..

[26] Jensen K.N., Deding D., Sørensen J.C., Bjarkam C.R. (2009). Long-Term Implantation of Deep Brain Stimulation Electrodes in the Pontine Micturition Centre of the Göttingen Minipig. Acta Neurochir..

[27] Langdale C.L., Grill W.M. (2016). Phasic Activation of the External Urethral Sphincter Increases Voiding Efficiency in the Rat and the Cat. Exp. Neurol..

[28] Foditsch E.E., Hoinoiu B., Janetschek G., Zimmermann R.P. (2014). Laparoscopic Placement of a Tined Lead Electrode on the Pudendal Nerve with Urodynamic Monitoring of Bladder Function during Electrical Stimulation: An Acute Experimental Study in Healthy Female Pigs. Springerplus.

[29] Keller E.E., Patras I., Hutu I., Roider K., Sievert K., Aigner L., Janetschek G., Lusuardi L., Zimmermann R., Bauer S. (2020). Early Sacral Neuromodulation Ameliorates Urinary Bladder Function and Structure in Complete Spinal Cord Injury Minipigs. Neurourol. Urodyn..

[30] Barone R. (2010). Comparative Anatomy of Domestic Mammals.

[31] Strauss I., Niederhoffer T., Giannotti A., Panarese A.M., Bernini F., Gabisonia K., Ottaviani M.M., Petrini F., Recchia F., Raspopovic S. (2020). The Q-PINE: A Quick-to-Implant Peripheral Intraneural Electrode. J. Neural Eng..

[32] Cai H., Morgan T., Pace N., Shen B., Wang J., Roppolo J.R., Horlen K., Khanwilkar P., Groat W.C., Tai C. (2019). Low Pressure Voiding Induced by a Novel Implantable Pudendal Nerve Stimulator. Neurourol. Urodyn..

[33] Bremer R.E., Barber M.D., Coates K.W., Dolber P.C., Thor K.B. (2003). Innervation of the Levator Ani and Coccygeus Muscles of the Female Rat. Anat. Rec..

[34] Kane D.D., Shott S., Hughes W.F., Kerns J.M. (2002). Motor Pudendal Nerve Characterization in the Female Rat. Anat. Rec..

[35] Pierce L.M., Reyes M., Thor K.B., Dolber P.C., Bremer R.E., Kuehl T.J., Coates K.W. (2003). Innervation of the Levator Ani Muscles in the Female Squirrel Monkey. Am. J. Obstet. Gynecol..

[36] Nyangoh Timoh K., Bessede T., Lebacle C., Zaitouna M., Martinovic J., Diallo D., Creze M., Chevallier J.-M., Darai E., Benoît G. (2017). Levator Ani Muscle Innervation: Anatomical Study in Human Fetus. Neurourol. Urodyn..

[37] Galindo R., Barba V., Dail W.G. (1997). The Sensory Branch of the Pudendal Nerve Is the Major Route for Adrenergic Innervation of the Penis in the Rat. Anat. Rec..

[38] Pastelín C.F., Zempoalteca R., Pacheco P., Downie J.W., Cruz Y. (2008). Sensory and Somatomotor Components of the “Sensory Branch” of the Pudendal Nerve in the Male Rat. Brain Res..

[39] Ambadkar P.M., Vyas D.M. (1981). Innervation of the Rat Preputial Gland. Cells Tissues Organs.

[40] Petrini F.M., Valle G., Strauss I., Granata G., Di Iorio R., D’Anna E., Čvančara P., Mueller M., Carpaneto J., Clemente F. (2019). Six-Month Assessment of a Hand Prosthesis with Intraneural Tactile Feedback: Hand Prosthesis. Ann. Neurol..

[41] Boretius T., Badia J., Pascual-Font A., Schuettler M., Navarro X., Yoshida K., Stieglitz T. (2010). A Transverse Intrafascicular Multichannel Electrode (TIME) to Interface with the Peripheral Nerve. Biosen. Bioelectron..

[42] Keung S.M. (2020). Characterizing Lower Urinary Tract Dysfunction in a Porcine Model of Spinal Cord Injury. Master’s Thesis.

